# Perceptions and experiences of undergraduate pharmacy students and alumni toward research after exposure to undergraduate research courses

**DOI:** 10.3389/fmed.2022.988908

**Published:** 2022-09-08

**Authors:** Banan Mukhalalati, Sara Elshami, Ola Adlan, Marwa Elshazly, Ahmed Awaisu, Derek Stewart, Daoud Al-Badriyeh, Feras Alali

**Affiliations:** College of Pharmacy, QU Health, Qatar University, Doha, Qatar

**Keywords:** pharmacy, research courses, significance, interest, confidence, outcomes, motivation, Theoretical Domains Framework (TDF)

## Abstract

**Introduction:**

Academic institutions have a duty to equip health professional students with the requisite research skills to ensure the implementation of evidence-based practice. This study aims to determine the perceptions of pharmacy students and alumni toward research after completing Undergraduate Research in Pharmacy Courses (URPCs) at the College of Pharmacy–Qatar University (CPH–QU).

**Methods:**

A cross-sectional survey was conducted. All CPH-QU alumni (*n* = 238), and all third- and fourth-year professional students who had completed at least one URPC at the time of conducting the study (*n* = 42) were approached. The questionnaire contained items relating to research experience and perceptions of significance, confidence in conducting research, actual and anticipated outcomes, and motivation for future research. A Theoretical Domains Framework informed the development of selected items.

**Results:**

The response rate was 72.1% (202/280); however, the usable rate was 95.5% (193/202). The participants gave positive responses relating to their perceptions of research significance {Median = 5.0 [Interquartile range (IQR) = 1.0], Minimum–Maximum = 1–5}, confidence in conducting research [Median = 4.0 (IQR = 1.0), Minimum–Maximum = 1–5], actual and anticipated outcomes [Median = 4.0 (IQR = 1.0), Minimum–Maximum = 1–5], and motivation for future research plans [Median = 4.0 (IQR = 1.0), Minimum–Maximum = 1–5]. The majority of participants perceived non-confidence in using data analysis software [72 (39.4% non-confidence)] and a high proportion of participants were non-confident in conducting data analysis [45 (24.6% non-confidence)]. More than half reported publishing at least one peer-reviewed article [99 (54.4% agreement)] from their courses and were highly motivated to consider post-graduate degrees in pharmacy [132 (73.3% agreement)].

**Conclusions:**

Incorporating URPCs into CPH–QU curriculum has potentially improved students and alumni's perceptions of research. Action is needed to improve confidence in different aspects of research.

## Introduction

Continuous advancement in healthcare requires the development of healthcare professionals committed to Evidence-Based Practice (EBP) (1). However, the successful integration of EBP into clinical practice is hindered by inadequate research knowledge, skills, experience, and confidence among healthcare professionals (2, 3). Therefore, research is a priority for many healthcare professional education programs (4).

Research is fundamental to evidence-based pharmacy practice (5), generating knowledge and advancing practices, establishing innovative roles and services, and improving health outcomes (6, 7). Given the significance of pharmacy research, it is critical to prepare pharmacy students and graduates with the requisite knowledge, skills, and willingness to conduct research and apply the findings to clinical practice (8, 9).

In the last few decades, pharmacy education around the globe has evolved significantly, with substantial advancements in curriculum, pedagogical methods, and instructional strategies (10, 11). The accreditation standards for the 2016 Accreditation Council for Pharmacy Education (ACPE) (12) and the 2018 Canadian Council for Accreditation of Pharmacy Programs (CCAPP) (13) highlight the importance of the achievement of specific research competencies by pharmacy students. These are outlined by the Task Force for the American College of Clinical Pharmacy (ACCP) on Research in the Professional Curriculum who emphasize eight key curricular competencies linked to research for students in pharmacy schools: identifying relevant problems; generating a hypothesis; designing a study; analyzing data; interpreting and applying findings; disseminating research findings; and applying regulatory and ethical principles (14).

Pharmacy colleges worldwide offer designated research tracks (15, 16), or directed research courses as core or elective requirements (17–19). In several studies, students indicated improved confidence when conducting research after completing a one-semester research course (17), and in applying knowledge in practice after completing an independent research elective course (20). Likewise, health professional students' early immersion in health-related research provides them with an opportunity to improve their knowledge, expertise, attitudes, and self-efficacy demonstrated by being able to define a problem, systematically gather data, think critically and analytically, and become lifelong learners (21). Furthermore, previous studies have found that health professional students' research experience is related to future professional and academic research endeavors (22, 23). The provision of adequate research facilities and supervision are also key requirements in preparing students for future practice (24).

There is a scarcity of research investigating pharmacists and pharmacy students' perspectives into research and the significance of research courses in the likely implementation of EBP in the Middle East. In Qatar, pharmacy practice is developing in all healthcare settings (25, 26), with a comparable advancement in pharmacists' engagement in health-related research as per the national requirement of pharmacy education (27). The College of Pharmacy at Qatar University (CPH–QU), accredited by the CCAPP, was founded in 2006 and represents its first and only pharmacy college. The focus at CPH–QU during their 5-year undergraduate degree program is on equipping students with research competencies *via* six Pharmacy Research Evaluation and Presentation Skills (PREP) courses, and two Undergraduate Research in Pharmacy Courses (URPCs). The two URPC courses are PHAR 445 (elective) and PHAR 545 (mandatory) in the third (P3) and the fourth (P4) professional years (28).

This study aims to examine the perceptions of pharmacy students and alumni of research following the completion of at least one of the URPCs. The specific objectives are to determine: (1) research experiences and perceptions of research significance; (2) perceptions of confidence in conducting pharmacy-related research; and (3) perceptions of the impact of participating in the URPCs on anticipated and actual outcomes, and their motivation for future research.

## Materials and methods

### Design and setting

The study was a cross-sectional design conducted at CPH–QU from August to December 2021. Ethics approval was obtained from the QU Institutional Review Board (QU-IRB 1562-EA/21).

### Study population

**“**The total number of CPH–QU alumni [i.e., from the first batch of graduates (2011) to the most recent batch of graduates (2021)], and all third- and fourth-year professional students who had completed at least one URPC was 287 (*n* = 244 alumni and 43 students). Raosoft^®^ online calculator was used to determine the minimum sample size of participants, excluding the 6 alumni and 1 student who conducted the face validity, and taking into consideration a 5% margin of error and confidence level of 95%. A sample of 162 participants was needed. However, the total eligible population (*n* = 280) was approached considering the possible non-response.“

### Undergraduate research in pharmacy courses

URPCs are two-credit 1-h courses [PHAR 445 (elective) and PHAR 545 (mandatory)] in P3 and P4 professional years, respectively (28). The courses provide students with an opportunity to conduct research under the supervision of a faculty member. Students are taught in the URPCs to comprehend and apply conceptual, theoretical, and empirical components of various research types within a wide area of pharmacy research. In addition to enhancing research skills, students are tutored in other scholarly-related activities such as scientific writing and presentation skills (i.e., poster and oral presentations), and are offered the opportunity to participate in research seminars, forums and conferences.

### Questionnaire development and validation

A self-administered questionnaire was developed and informed by previous studies (6, 17, 24, 29–34). Specific items were guided by the Theoretical Domains Framework (TDF), an integrated framework of 33 behavior change theories described in 14 domains of behavior determinants (influences) (35). Specific domains of interest were knowledge, skills, belief in capabilities, professional role/identity, emotions, beliefs in consequences, intentions, and goals. Incorporating theory into research improves the robustness, relevance, and impact of findings (36).

To determine the likely content validity, the first draft was shared with 15 local, regional, and international pharmacy education and practice experts, and the questionnaire was developed over two stages. The first involved a qualitative evaluation and review of the content relevance, representativeness, and technical quality of each item. The second round involved utilizing an item Content Validity Index (i-CVI) and Scale-Content Validity/Average (S-CVI/Ave) for item-reduction purposes, which revealed acceptable scores and, hence, no item was removed. Face validity was conducted by purposefully selecting seven participants (6 CPH–QU alumni and 1 current student who had completed at least one URPC. These were then requested to consider the representativeness of each item to the actual experience of the target population. This led to the removal of three questionnaire items to reduce redundancy, and the rephrasing of several items to enhance readability and understandability.

The final version of the questionnaire comprised 68 items, divided into four sections: demographics and professional characteristics (11 items); research experience (5 items) and perceptions of research significance (11 items; 5-point Likert scale [1 = strongly disagree to 5 = strongly agree] based on knowledge, professional role/identity, and emotion domains of TDF); confidence in conducting research (19 items; 5-point Likert scale [1 = not confident at all to 5 = extremely confident] based on self-efficacy and skills domains of TDF); perceptions of impact (7 items and an open-ended item) and anticipated outcomes (4 items based on belief in consequences domain of TDF; 5-point Likert scale and an open-ended item); and motivation for future research (9 items; 5-point Likert scale, based on intentions and goals domain of TDF) (See Supplementary material 1 for the mapping of questionnaire items to TDF).

### Data collection procedure

The personal e-mail addresses of the study population were retrieved from a CPH–QU database, with each invited to complete an anonymous, self-administered questionnaire through a web-link at SurveyMonkey^®^ (Survey Monkey Inc., San Mateo, California, USA). A detailed description of the study (e.g., objectives, data collection procedure, confidentiality and anonymity measures, and voluntary nature of participation) was provided. The completion of the questionnaire implied an informed consent to participate. The questionnaire link was open from early November to late December 2021, and three reminders were sent.

### Statistical analysis

Descriptive and inferential statistics were performed using IBM Statistical Package for Social Sciences (IBM SPSS^®^ 27 software). Non-parametric statistics were used to compare different subgroups in terms of the overall perceptions of research significance, confidence in research, research outcomes, and motivation for future research. Sub-group analyses targeted having a post-graduate degree (yes or no), practice setting (hospital-based, non-hospital based or not practicing), years of work experience (0, <1, 1–5, 6–10 or >10 years), URPC research discipline (clinical pharmacy, pharmaceutical sciences or both), additional research projects undertaken (yes or no), and whether participating in publication and/or poster/oral presentations (yes or no). Mann-Whitney U and Kruskal-Wallis H tests were used to compare variables with two and more than two categories, respectively. The level of statistical significance was set *a priori* at *p* < 0.05. The questionnaire scales were tested for internal consistency reliability (Cronbach's alpha), considering α ≥ 0.7 as the minimum satisfactory value.

## Results

### Demographics and professional characteristics

The response rate was 72.1% (*n* = 202 of 280); however, the usable rate was 95.5% (*n* = 193 of 202), after removing respondents who filled the demographic section only. The questionnaire sections were not filled completely by all the 193 participants; hence, each section was analyzed separately to maintain the adequacy of responses. Therefore, different numbers of participants were reported in each section. Participants' demographic and professional characteristics are presented in Table 1. The majority were CPH–QU alumni [151 (78.2%)] who graduated between 2014 and 2019 [90 (60.4%]), acquired a post-graduate degree [95 (62.9%)], predominantly from the CPH–QU PharmD program [66 (69.5%)]. The majority of the CPH–QU alumni were full-time employees [100 (85.5%)], working in governmental hospitals [56 (37.33%)] for 1–5 years [65 (43.0%)].

**Table 1:** Demographics and professional characteristics of participants.

**Demographics and professional characteristics**	**Frequency (%)**
**Age**	***N** **=*** **193**
Less than 20 years	1 (0.5)
20 – 24 years	74 (38.3)
25 – 29 years	69 (35.8)
30 years or more	49 (25.4)
**Current status at the CPH**	***N** **=*** **193**
3rd professional year undergraduate student	18 (9.3)
4th professional years undergraduate student	24 (12.4)
Pharmacy graduate (BSc and above)	151 (78.2)
**Year of graduation**	***N** **=*** **149**
2011–2013	24 (16.1)
2014–2016	44 (29.5)
2017–2019	46 (30.9)
2020–2021	35 (23.5)
**Post-graduate degree**	***N** **=*** **151**
Yes	95 (62.9)
No	56 (37.1)
Post-graduate degree*	***N** **=*** **95**
MSc degree	28 (29.5)
PharmD degree	66 (69.5)
Other (e.g., MPH, PGY1, diploma in non-pharmacy discipline, etc.)	16 (16.8)
**Current enrolment in a postgraduate program**	***N** **=*** **151**
Yes	30 (19.9)
No	121 (80.1)
Current professional practice setting*	***N** **=*** **152**
Not currently practicing/working	34 (22.4)
Community pharmacy	9 (5.9)
Governmental/public hospital	56 (36.8)
Research institute	15 (9.9)
Semi-governmental/public hospital	13 (8.6)
Other (pharmaceutical industry, academic institute, ministry of public health, or primary health care setting)	25 (16.4)
**Type of employment**	***N** **=*** **117**
Full time	100 (85.5)
Part time	5 (4.3)
Other (temporary, volunteering, or training)	12 (10.3)
**Years of work experience in pharmacy-related practice**	***N** **=*** **151**
None	29 (19.2)
<1 year	27 (17.9)
1–5 years	65 (43.0)
6–10 years	28 (18.5)
More than 10 years	2 (1.3)

^*^A multiple response item.

### Participants' perceptions of research after completing the undergraduate research in pharmacy courses

#### Perceptions of research experience and significance

Table 2 presents the participants' research experience and background. Almost half [101 (52.3%)] were involved in both URPCs (i.e., PHAR 445 and PHAR 545). The majority [118 (61.1%)] had participated in additional research projects (other than URPCs) during their undergraduate pharmacy degree, primarily *via* the Undergraduate Research Experience Program (UREP) of the Qatar National Research Fund [107 (91.5%)].

**Table 2:** Participants' research experience.

**Item**	**Frequency (%)**
**Undergraduate Research in Pharmacy Courses (URPC)s taken**	***N** **=*** **193**
The elective research course in the third professional year (PHAR445)	41 (21.2)
The elective research course in the fourth professional year (PHAR545)	34 (17.6)
Both PHAR445 and PHAR545	101 (52.3)
I cannot recall	17 (8.8)
**The focus of the URPC**	***N** **=*** **193**
Clinical pharmacy and practice research only	93 (48.2)
Pharmaceutical sciences research only	25 (13.0)
Both clinical and pharmaceutical sciences research	73 (37.8)
I cannot recall	2 (1.0)
**Additional research experience obtained during studying the BSc degree**	***N** **=*** **193**
Yes	118 (61.1)
No	69 (35.8)
I cannot recall	6 (3.1)
**Number of the additional experience obtained during studying the BSc degree**	***N** **=*** **117**
1	82 (70.1)
2	24 (20.5)
≥ 3	11 (9.4)
**Type of the additional research experience obtained during studying the BSc degree**	*N =* 117
Undergraduate Research Experience Program (UREP)	107 (91.5)
Other (e.g., Research internship)	10 (8.5)

Participants' perceptions regarding the significance of, and interest in, pharmacy-related research are presented in Table 3 {Median = 5.0 [Interquartile range (IQR) = 1.0], Minimum–Maximum = 1–5}. The internal consistency of this scale was = 0.898 (Supplementary material 2). The majority expressed agreement levels of more than 80% with the significance of URPCs and their interest in research, as indicated by responses to most items. A Mann-Whitney U test identified that those who participated in projects additional to URPC showed a significantly statistically higher perception of research importance and interest than those who did not (*U* = 3,103.50, *p* = *0.013*) (Supplementary material 3).

**Table 3:** Participants' perceptions of research after completing the undergraduate research in pharmacy courses.

	**Frequency (%)**						
**Item I believe that**	**Strongly disagree**	**Disagree**	**Neutral**	**Agree**	**Strongly agree**	**Missing *N***	**Median (IQR)**
**Significance of, and interest in research**							
Conducting research in the field of pharmacy is valuable in my practice	10 (5.3)	0 (0.0)	6 (3.2)	41 (21.9)	130 (69.5)	6	5 (1)
Research is important for advancements in the pharmacy profession	10 (5.3)	0 (0.0)	4 (2.1)	31 (16.6)	142 (75.9)	6	5 (0)
Developing varying research skills and competencies by incorporating them into various undergraduate research-related courses is important	10 (5.3)	0 (0.0)	3 (1.6)	46 (24.6)	128 (68.4)	6	5 (1)
Research courses or projects are an important component of an undergraduate pharmacy curriculum	10 (5.4)	0 (0.0)	4 (2.2)	33 (17.7)	139 (74.7)	7	5 (1)
I understand the distinctions between different kinds of pharmacy-related research (e.g., practice-based research and pharmaceutical science research)	10 (5.3)	4 (2.1)	17 (9.1)	77 (41.2)	79 (42.2)	6	4 (1)
I understand how practice-based (e.g., clinical) research and bench-side (e.g., lab-based) research in pharmacy complement each other	11 (5.9)	3 (1.6)	12 (6.4)	76 (40.6)	85 (45.5)	6	4 (1)
I am interested in further developing my knowledge and skills in pharmacy-related research	11 (5.9)	0 (0.0)	5 (2.7)	57 (30.5)	114 (61.0)	6	5 (1)
I am satisfied with my involvement in pharmacy-related research activities	13 (7.0)	20 (10.7)	25(13.4)	62 (33.2)	67 (35.8)	6	4 (2)
I am interested in conducting pharmacy-related research	8 (4.3)	0 (0.0)	9 (4.8)	59 (31.6)	111 (59.4)	6	5 (1)
Incorporating research courses into undergraduate curriculum is an overload for students and unnecessary*	79 (42.2)	66 (35.3)	12 (6.4)	16 (8.6)	14 (7.5)	6	4 (1)
Allocating time to conduct research is a waste if it does not advance my future career *	72 (38.5)	62 (33.2)	17 (9.1)	20 (10.7)	16 (8.6)	6	4 (2)
**Confidence in conducting research**							
	**Frequency (%)**						
**Item I am confident in my ability to**	**Not at all confident**	**Not very confident**	**Moderately confident**	**Very confident**	**Extremely confident**	**Missing** ***N***	**Median (IQR)**
Critically review or appraise published literature	0 (0.0)	4 (2.2)	50 (27.3)	88 (48.1)	41 (22.4)	10	4 (1)
Identify gaps in current knowledge\literature	0 (0.0)	9 (4.9)	74 (40.4)	66 (36.1)	34 (18.6)	10	4 (1)
Generate a relevant research idea/questions and testable hypothesis	1 (0.5)	13 (7.1)	68 (37.2)	72 (39.3)	29 (15.8)	10	4 (1)
In my ability to develop research aims and objectives	0 (0.0)	6 (3.3)	52 (28.4)	86 (47.0)	39 (21.3)	10	4 (1)
Write a research proposal or protocol	7 (3.8)	19 (10.4)	58 (31.7)	69 (37.7)	30 (16.4)	10	4 (1)
Determine the most appropriate study designs or methodology	6 (3.3)	20 (10.9)	65 (35.5)	67 (36.6)	25 (13.7)	10	4 (1)
Ensure adherence to ethical standards	1 (0.5)	7 (3.8)	43 (23.5)	83 (45.4)	49 (26.8)	10	4 (2)
Acquire ethics approvals (e.g. human or animal ethics approval, biohazard approval)	7 (3.8)	32 (17.6)	52 (28.6)	55 (30.2)	36 (19.8)	11	4 (1)
Select data collection tools/methods	1 (0.5)	21 (11.5)	61 (33.5)	71 (39.0)	28 (15.4)	11	4 (1)
Conduct data collection	1 (0.5)	6 (3.3)	45 (24.6)	84 (45.9)	47 (25.7)	10	4 (2)
Select data analyses tools/instruments	5 (2.7)	24 (13.2)	59 (32.4)	63 (34.6)	31 (17.0)	11	4 (1)
Conduct data analyses appropriately	9 (4.9)	36 (19.7)	64 (35.0)	52 (28.4)	22 (12.0)	10	3 (1)
In data entry, storage and management of collected data using database	5 (2.7)	22 (12.0)	56 (30.6)	70 (38.3)	30 (16.4)	10	4 (1)
Use data analysis software	25 (13.7)	47 (25.7)	57 (31.1)	39 (21.3)	15 (8.2)	10	3 (2)
Summarize results using tables, or charts, or quotes, or others as relevant	2 (1.1)	10 (5.5)	51 (27.9)	74 (40.4)	46 (25.1)	10	4 (2)
In my ability to interpret the study results	0 (0.0)	4 (2.2)	49 (26.9)	83 (45.6)	46 (23.8)	11	4 (2)
Develop appropriate conclusions based on results from research	0 (0.0)	2 (1.1)	51 (27.9)	82 (44.8)	48 (26.2)	10	4 (2)
Prepare a written report about a completed research project	1 (0.5)	6 (3.3)	48 (26.2)	84 (45.9)	44 (24.0)	10	4 (1)
Prepare an oral and or poster presentation of a completed research project for a scientific forum	0 (0.0)	4 (2.2)	37 (20.2)	73 (39.9)	69 (37.7)	10	4 (1)
**Outcomes of participation in the undergraduate research in pharmacy courses**							
	**Frequency (%)**						
**Item I believe that**	**Strongly disagree**	**Disagree**	**Neutral**	**Agree**	**Strongly agree**	**Missing** ***N***	**Median (IQR)**
Undertaking undergraduate research project(s) provided me with professional satisfaction	7 (3.9)	6 (3.3)	22 (12.2)	75 (41.4)	71 (39.2)	12	4 (1)
My undergraduate research has a potential contribution to literature	7 (3.9)	8 (4.4)	28 (15.5)	83 (45.9)	55 (30.4)	12	4 (1)
Publishing the findings of undergraduate research in a peer-reviewed journal is an excellent source of recognition	4 (2.2)	1 (0.6)	7 (3.9)	77 (42.5)	92 (50.8)	12	5 (1)
Participating in undergraduate research was beneficial to my professional career	6 (3.1)	4 (2.1)	18 (9.3)	75 (38.9)	78 (40.4)	12	4 (1)
**Motivations for future research plans**							
	**Frequency (%)**						
**Item I am motivated (was motivated) to**	**Strongly disagree**	**Disagree**	**Neutral**	**Agree**	**Strongly agree**	**Missing** ***N***	**Median (IQR)**
Pursue a post-graduate pharmacy-related research degree after conducting the undergraduate research	1 (0.6)	12 (6.7)	35 (19.4)	64 (35.6)	68 (37.8)	13	4 (2)
Pursue a pharmacy-related research career after conducting the undergraduate research	0 (0.0)	16 (8.9)	35 (19.4)	70 (38.9)	59 (32.8)	13	4 (2)
Advance my knowledge and skills in conducting pharmacy-related research	0 (0.0)	1 (0.6)	14 (7.8)	83 (46.1)	82 (45.6)	13	4 (1)
Participate in pharmacy-related research throughout my pharmacy career	0 (0.0)	1 (0.6)	13 (7.2)	81 (45.0)	85 (47.2)	13	4 (1)
Advance in my pharmacy career through participating in research	0 (0.0)	1 (0.6)	18 (10.0)	76 (42.2)	85 (47.2)	13	4 (1)
I believe that continuously advancing my knowledge and skills in conducting pharmacy-related research is a waste of time *	74 (41.3)	53 (29.6)	8 (4.5)	22 (12.3)	22 (12.3)	14	4 (3)
Apply for research grants	1 (0.6)	6 (3.4)	37 (20.8)	72 (40.4)	62 (34.8)	15	4 (1)
Publish pharmacy-related research papers in order to advance the pharmacy field	0 (0.0)	3 (1.7)	15 (8.3)	84 (46.7)	78 (43.3)	13	4 (1)
Present more pharmacy-related research oral/poster presentations at conferences	1 (0.6)	3 (1.7)	25 (13.9)	72 (40.0)	79 (43.9)	13	4 (1)

^*^A reversed median score item.

#### Perceptions of confidence in conducting research

Perceptions of their confidence in conducting research are presented in Table 3 [Median = 4.0 (IQR = 1.0), Minimum–Maximum = 1–5]. The internal consistency of this scale was 0.948 (Supplementary material 2). Over three-quarters were confident (i.e., very confident or extremely confident) in their ability to prepare an oral or poster presentation [142 (77.6%)] and in ensuring adherence to ethical standards [132 (72.2%)]. More than a quarter of the participants indicated moderate confidence in almost all items (16/19 items). Conversely, a large proportion reported a lack of confidence (i.e., not confident at all and not very confident) in using data analysis software [72 (39.4%)], and in conducting data analyses [45 (24.6%)]. Kruskal Wallis H test showed a statistically significant difference in confidence in conducting research between those alumni who worked in hospital-based practices and those in non-hospital-based practices (*H* = *6.783, p* = 0.034*)* (Supplementary material 3).

#### Perceptions of research outcomes

Reported research outcomes following completion of URPCs are provided in Table 4. Many indicated publish at least one peer-reviewed article (99 [54.4%]). Mann-Whitney U test indicated that participants who had published at least one peer-reviewed article had a significantly higher perception of their confidence in conducting research than those who did not (*U* = 3209.0, *p* = 0.025) (Supplementary material 3). Presenting a poster from the URPCs at an international conference was associated with a significantly higher level of confidence in conducting research (U = 2982.0, *p* = *0.007*) (Supplementary material 3).

**Table 4:** Participants' reported outcomes after taking the undergraduate research in pharmacy courses (URPC)s.

**Item**	**Frequency (%)**
**Publishing at least one peer-reviewed article from my URPC**	***N** **=*** **182**
Yes	99 (54.4)
No	79 (43.4)
I cannot recall	4 (2.2)
**Presenting at least one poster from my URPC in a local conference**	***N** **=*** **182**
Yes	135 (74.2)
No	43 (23.6)
I cannot recall	4 (2.2)
**Presenting at least one poster from my URPC in an international conference**	***N** **=*** **182**
Yes	78 (42.9)
No	98 (53.8)
I cannot recall	6 (3.3)
**Presenting at least one podium/oral presentation from my URPC in a local conference**	***N** **=*** **181**
Yes	90 (49.7)
No	81 (44.8)
I cannot recall	10 (5.5)
**Presenting at least one podium/oral presentation from my URPC in an international conference**	***N** **=*** **182**
Yes	36 (19.8)
No	140 (76.9)
I cannot recall	6 (3.3)
**Submitting at least one abstract from my URPC for a conference**	***N** **=*** **182**
Yes	121 (66.5)
No	54 (29.7)
I cannot recall	7 (3.8)

Table 3 gives participants' perceptions of the actual and anticipated outcomes of participation in URPCs [Median = 4.0 (IQR = 1.0), Minimum–Maximum = 1–5]. The internal consistency of this scale was 0.857 (Supplementary material 2). The majority [169 (93.3%)] considered the publication of URPCs findings in a peer-reviewed journal as an excellent source of recognition. Mann-Whitney U demonstrated that undertaking other research projects in addition to the URPCs was associated with a significantly higher perception of outcomes of participating in URPCs (U = 2740.0, *p* = 0.006) (Supplementary material 3).

#### Perceptions of the motivations for future research plans

Participants' perceptions toward motivations for future research plans are presented in Table 3 [Median = 4.0 (IQR = 1.0), Minimum–Maximum = 1–5]. The internal consistency of this scale was 0.832 (Supplementary material 2). The majority of participants reported agreement with items related to participants' motivations for future research (i.e., agree and strongly agree, ranging from 71.7 to 92.2%). The largest proportion of participants reported agreements with motivation to participate in pharmacy-related research [166 (92.2%)] and with motivation to advance knowledge and skills in conducting research [165 (91.7%)]. A Mann-Whitney U identified a statistically significant association between those undertaking other research projects in addition to URPCs and higher perceptions of motivation for future career plans (*U* = 2,727.5, *p* = 0.004) (Supplementary material 3).

## Discussion

The majority of participants expressed agreement with the significance of conducting pharmacy profession research, and the importance of its incorporation into undergraduate pharmacy curricula. This is similar to results obtained by Kritikos et al. (6) who noted that around three-quarters of pharmacy students at the University of Sydney recognized the significance of incorporating research-related activities in their undergraduate curriculum (6). A lack of formal research courses in the curriculum was largely considered a barrier to research activity engagement among dental hygiene students (37). Several factors negatively impact health professional students' perceptions of research significance, such as the probability of research projects yielding insignificant results despite the time and effort invested in them (30, 38). Contrary to a Saudi Arabian study (30), the majority of participants believed research to be essential and not a waste of time regardless of career advancement.

An important finding of this study, consistent with results found in other studies (6, 32) was the considerable proportion (>25%) of participants who reported moderate confidence to various aspects of research surveyed in the questionnaire items. Moreover, the present study also found that the majority of participants perceived non-confidence in their ability to use data analysis software and a high proportion of participants were non-confident in conducting data analysis. aligning with results reported from a number of previous studies on pharmacy students (32, 39), and students of other health professions (40, 41). Improving students' comprehension and application of statistical tests is an area for improvement. Participants showed more confidence in their ability to prepare oral or poster presentations of a completed research project, a result which may be attributed to URPC-based orientation sessions around these skills, in addition to being exposed to a variety of research evaluations and presentation skills in several courses and journal clubs within the curriculum. Similarly, pharmacy students in the United States indicated that participation in elective research courses resulted in their improved engagement with research-related activities, such as poster and podium presentations and peer-reviewed publications (42).

CPH–QU alumni working in hospital settings perceived themselves to have a significantly lower rate of confidence in conducting research than those who worked in non-hospital settings (e.g., academia and research institutes). This finding is in concordance with studies by Awaisu et al. (27) investigating hospital pharmacists in Qatar, who lacked competence and confidence in several aspects of research, such as developing research protocols, critically appraising literature, determining sample size, and interpreting research findings. Conducting research is neither part of the job description of a hospital pharmacist, nor a requirement for preregistration in Qatar or many other countries (32); hence, engagement in research activities is not considered a priority.

The current study found that participants placed a high value on publishing their undergraduate research in peer-reviewed journals, which they considered to be an excellent source of recognition. A similar finding was reported in a study conducted in the United States where more than 90% of PharmD students believed that publications provided a sense of recognition and excellence, with the authors noting that research participation facilitates career advancement (43). A study conducted among research advisors at Ambo University, Ethiopia, demonstrated that there is an absence of knowledge transfer through publication and presentation which was a potential outcome of poor undergraduate pharmacy students' research projects (44). It has also been reported that inadequate research mentoring can hinder student research activity and associated outputs (45).

This study showed that the majority of participants were motivated to pursue a post-graduate pharmacy-related research degree and to work in a research-related career, similar to a recent study in Saudi Arabia (30). Conversely, a study conducted in Canada demonstrated that few pharmacy students were interested in pursuing postgraduate research due to a lack of interest in a research career (46). In that regard, Johnson et al. (47) suggested that early exposure to research during the undergraduate study is necessary for pharmacy graduates to show a preference for a research-related career (47). In a cross-sectional study of medical and allied health professional students, Bovijn et al. (23) found there to be greater interest in future research among those who had previous or current research experience (23). Furthermore, Overholser et al. (48) and Surratt et al. (15) argued that enhancing pharmacy students' perception of research significantly improves their motivation to conduct research studies in their professional practices (15, 48).

TDF was used in this study to provide a focus on potential research behavior influences. Although participants self-reported high levels of knowledge and understanding regarding the significance of research to the pharmacy, their beliefs about capabilities (i.e., lack of confidence in their abilities to perform different research processes) might benefit from tailored behavior change interventions. Indeed, others have suggested teaching strategies that increase student conviction in their ability to successfully perform research tasks (49). Moreover, it has been shown that inadequate training in several research competencies (e.g., research methodology, and data analysis) is the most significant obstacle to undertaking high-quality research in developing countries (50). This suggests that teaching research as a mandatory course would eliminate this challenge. Cailor et al. (17) claimed that research courses offered as electives might only be taken by students who were already interested in pursuing a research-based career (17). As today's healthcare practice is an integral aspect of evidence-based health care, research education and training should be mandatory in undergraduate health professions education, including pharmacy.

## Strengths and limitations

The strengths of this study include the steps taken in pretesting the questionnaire, and the inclusion of TDF in several items with the aim of enhancing internal validity. The response rate was relatively high, minimizing the potential for non-response bias (51). Nevertheless, the findings of the current study should be interpreted with caution due to the relatively small population size and the potential for underpowered statistical comparisons. Also, the questionnaire evaluation did not include other types of validity tests (e.g., construct validity). There may have been an element of recall bias, particularly for the batch of alumni who graduated the earliest. While the findings may not be generalizable to other settings and populations, many of the findings do resonate with the literature.

## Further research

There would be merit in conducting longitudinal studies with similar populations and colleges in other similar Arab countries while comparing perceptions of students who participated in elective research courses and those who did not participate. Moreover, it might be useful to compare how students perceive research from the alumni population, which was not feasible in this study due to the small sample student population. Furthermore, qualitative research would provide an in-depth understanding of the key findings. Additional psychometric tests (e.g., construct and criterion validities) may be warranted to provide further validation of the designed questionnaire.

## Conclusions

Incorporating research courses into the undergraduate pharmacy curriculum at CPH–QU has potentially improved students and alumni's perceptions of research significance, their interest and confidence in conducting pharmacy-related research, and their motivation for perusing a research-related career. Further action is needed to improve confidence in different aspects of pharmacy-related research.

## Data availability statement

The raw data supporting the conclusions of this article will be made available by the authors, without undue reservation.

## Ethics statement

Ethics approval was obtained from the QU Institutional Review Board (QU-IRB 1562-EA/21). The patients/participants provided their written informed consent to participate in this study.

## Author contributions

All authors listed have made a substantial, direct, and intellectual contribution to the work and approved it for publication.

## Funding

This research was supported by the (Qatar University internal grant) under Grant (QUST-2- CPH -2021-213). Open access funding is provided by Early Career Researcher Award (ECRA03-001-3-001) and Qatar National Research Fund.

## Conflict of interest

The authors declare that the research was conducted in the absence of any commercial or financial relationships that could be construed as a potential conflict of interest.

## Publisher's note

All claims expressed in this article are solely those of the authors and do not necessarily represent those of their affiliated organizations, or those of the publisher, the editors and the reviewers. Any product that may be evaluated in this article, or claim that may be made by its manufacturer, is not guaranteed or endorsed by the publisher.
