# Supplementary material for: Perceptions and experiences of undergraduate pharmacy students and alumni toward research after exposure to undergraduate research courses
Source: Front Med (Lausanne). 2022 Sep 8;9:988908. doi: 10.3389/fmed.2022.988908 (PMC9492970; doi:10.3389/fmed.2022.988908)
Supplement: Supplementary file 1 [file Table_1.pdf]

Supplementary Materials

Supplementary Material 1. Mapping Items of the Questionnaire to the Theoretical Domain Framework (TDF) Domains and Constructs

TDF domain	TDF construct	Examples from the questionnaire
Knowledge	Knowledge/ knowledge about the condition	I believe that I understand the distinctions between different kinds of pharmacy-related research (e.g., practice-based research, basic pharmaceutical science research, and applied translational research).
		I believe that I understand how practice-based (e.g., clinical) research and bench-side (e.g., lab-based) research in pharmacy complement each other.
Professional role and identity	Professional role/ identity	I believe that conducting research in the field of pharmacy is valuable in my practice.
		I believe that research is important for advancements in the pharmacy profession
Emotion	Stress/ Anxiety/ Burn-out/ Cognitive overload/ tiredness/ Negative affect/ Regret	I believe that incorporating research courses into undergraduate curriculum is an overload for students and unnecessary.
		I believe that allocating time to conduct research is a waste if it does not advance my future career.
Beliefs about capabilities, skills	Self-efficacy/ Self-professional confidence/ Perceived competence/ Skills	I am confident in my ability to critically review or appraise published literature.
		I am confident in my ability to conduct data analyses appropriately.
Beliefs about consequences	Outcome expectation/ Consequents/ Beliefs	I believe that my undergraduate research has a potential contribution to literature
		I believe that undertaking undergraduate research project(s) provided me with professional satisfaction.
		I believe that publishing the findings of undergraduate research in a peer-reviewed journal is an excellent source of recognition.
Intentions and goals	Intentions/ Goals	I am motivated (or was motivated) to pursue a post-graduate pharmacy-related research degree after conducting the undergraduate research.
	Proximal and distal goals	I am motivated to publish pharmacy-related research papers in order to advance the pharmacy field.

Supplementary Material 2. Reliability Statistics

2.1 Perceptions of Research Experience and Significance

**Scale Statistics**				
Cronbach's Alpha	Mean	Variance	Std. Deviation	N of items
.898	46.86	68.056	8.250	11

Item and item-total Statistics

	Mean	Std. Deviation	Scale Mean if Item Deleted	Scale Variance if Item Deleted	Corrected Item-Total Correlation	Cronbach's Alpha if Item Deleted	N
I believe that conducting research in the field of pharmacy is valuable in my practice.	4.50	.982	42.36	54.664	.856	.877	186
I believe that research is important for advancements in the pharmacy profession.	4.58	.968	42.28	54.767	.862	.877	186
I believe that developing varying research skills and competencies by incorporating them into various undergraduate research-related courses is important.	4.51	.966	42.35	54.479	.887	.876	186
I believe that research courses or projects are an important component of an undergraduate pharmacy curriculum.	4.56	.969	42.30	54.577	.876	.876	186
I believe that I understand the distinctions between different kinds of pharmacy-related research (e.g., practice-based research, basic pharmaceutical science research, and applied translational research).	4.12	1.035	42.74	55.482	.746	.883	186
I believe that I understand how practice-based (e.g., clinical) research and bench-side (e.g., lab-based) research in pharmacy complement each other.	4.18	1.043	42.68	54.866	.783	.881	186

I believe that I am interested in further developing my knowledge and skills in pharmacy-related research.	4.40	1.005	42.46	54.855	.819	.879	186
I believe that I am satisfied with my involvement in pharmacy-related research activities.	3.80	1.231	43.06	57.974	.457	.901	186
I believe that I am interested in conducting pharmacy-related research.	4.41	.927	42.45	55.838	.819	.880	186
Section_B2_10_recoded	3.97	1.230	42.89	63.783	.141	.920	186
Section_B2_11_recoded	3.83	1.282	43.03	63.691	.133	.922	186

2.2 Perceptions of Confidence in Conducting Research

Scale Statistics

Cronbach's Alpha	Mean	Variance	Std. Deviation	N of items
.948	69.84	154.889	12.445	19

Item and item-total Statistics

	Mean	Std. Deviation	Scale Mean if Item Deleted	Scale Variance if Item Deleted	Corrected Item-Total Correlation	Cronbach's Alpha if Item Deleted	N
I am confident in my ability to critically review or appraise published literature.	3.90	.758	65.94	143.204	.613	.947	179
I am confident in my ability to identifying gaps in current knowledge\literature.	3.67	.826	66.17	141.882	.626	.946	179
I am confident in my ability to generate a relevant research idea/questions and testable hypothesis.	3.63	.861	66.21	141.325	.626	.946	179
I am confident in my ability to develop research aims and objectives.	3.87	.782	65.97	140.825	.725	.945	179
I am confident in my ability to write a research proposal or protocol.	3.52	1.013	66.32	137.623	.683	.946	179
I am confident in my ability to determine the most appropriate study designs or methodology.	3.46	.979	66.37	137.303	.725	.945	179

I am confident in my ability to ensure adherence to ethical standards.	3.94	.839	65.89	139.713	.729	.945	179
I am confident in my ability to acquire ethics approvals (e.g. human or animal ethics approval, biohazard approval).	3.45	1.107	66.39	137.442	.625	.947	179
I am confident in my ability to select appropriate data collection tools/methods.	3.58	.904	66.26	137.125	.800	.944	179
I am confident in my ability to conduct data collection.	3.93	.828	65.91	139.677	.742	.945	179
I am confident in my ability to select data analyses tools/instruments (e.g., questionnaires or machines).	3.50	1.013	66.34	135.033	.800	.943	179
I am confident in my ability to conduct data analyses appropriately.	3.23	1.039	66.60	137.398	.674	.946	179
I am confident in my ability in data entry, storage and management of collected data using database.	3.55	.978	66.29	138.252	.682	.946	179
I am confident in my ability to use data analysis software.	2.85	1.144	66.99	136.618	.634	.947	179
I am confident in my ability to summarize results using tables, or charts, or quotes, or others as relevant.	3.83	.902	66.01	138.916	.713	.945	179
I am confident in my ability to interpret the study results.	3.94	.780	65.90	141.619	.682	.946	179
I am confident in my ability to develop appropriate conclusions based on results from research.	3.96	.767	65.88	141.726	.689	.946	179
I am confident in my ability to prepare a written report about a completed research project.	3.90	.822	65.94	140.856	.685	.946	179
I am confident in my ability to prepare an oral and or poster presentation of a completed research project for a scientific forum (e.g. conference or symposium).	4.13	.807	65.71	142.320	.619	.947	179

2.3 Perceptions of Research Outcomes

Scale Statistics

Cronbach's Alpha	Mean	Variance	Std. Deviation	N of items
.857	16.61	9.750	3.122	4

Item and item-total Statistics

	Mean	Std. Deviation	Scale Mean if Item Deleted	Scale Variance if Item Deleted	Corrected Item-Total Correlation	Cronbach's Alpha if Item Deleted	N
I believe that undertaking undergraduate research project(s) provided me with professional satisfaction.	4.09	.996	12.52	5.317	.749	.798	181
I believe that my undergraduate research has a potential contribution to literature.	3.94	.993	12.67	5.678	.652	.841	181
I believe that publishing the findings of undergraduate research in a peer-reviewed journal is an excellent source of recognition.	4.39	.786	12.22	6.362	.699	.825	181
I believe that participating in undergraduate research was beneficial to my professional career.	4.19	.942	12.43	5.624	.725	.808	181

2.4 Perceptions of the Motivations for Future Research Plans

Scale Statistics

Cronbach's Alpha	Mean	Variance	Std. Deviation	N of items
.832	37.44	26.805	5.177	9

Item and item-total Statistics

	Mean	Std. Deviation	Scale Mean if Item Deleted	Scale Variance if Item Deleted	Corrected Item-Total Correlation	Cronbach's Alpha if Item Deleted	N
I am motivated (or was motivated) to pursue a post-graduate pharmacy-related research degree after conducting the undergraduate research.	4.02	.947	33.42	20.642	.612	.806	177

I am motivated (or was motivated) to pursue a pharmacy-related research career after conducting the undergraduate research.	3.94	.940	33.50	21.070	.562	.812	177
I am motivated to advance my knowledge and skills in conducting pharmacy-related research.	4.36	.652	33.08	21.698	.770	.797	177
I am motivated to participate in pharmacy-related research throughout my pharmacy career.	4.38	.648	33.06	21.508	.812	.794	177
I am motivated to advance in my pharmacy career through participating in research.	4.36	.685	33.08	21.362	.785	.794	177
Section_D_6_recoded	3.75	1.421	33.69	24.997	-.015	.916	177
I am motivated to apply for research grants.	4.06	.860	33.38	20.998	.642	.803	177
I am motivated to publish pharmacy-related research papers in order to advance the pharmacy field.	4.32	.700	33.12	21.371	.764	.795	177
I am motivated to present more pharmacy-related research oral/poster presentations at conferences.	4.25	.801	33.19	20.974	.707	.797	177

Supplementary Material 3. Participants' overall perceptions of research significance, confidence in research, research outcomes, and motivation for future research according to research experience and professional characteristics

Item	Subgroups	Number of participants	Mean Rank	Sum of Ranks	Significance
**Additional degree**					
Research Experience and Significance	Yes	94	76.79	7218.00	U= 2135.00 P= 0.14
	No	52	67.56	3513.00	
Confidence in Conducting Research	Yes	93	74.53	6931.00	U=1997.00 P= 0.19
	No	49	65.76	3222.00	
Research Outcomes	Yes	92	72.75	6693.00	U= 2093.00 P= 0.46
	No	49	67.71	3318.00	
Motivations for Future Research Plans	Yes	91	73.84	6719.50	U= 1925.50 P= 0.14
	No	49	64.30	3150.50	
**Practice setting**					
Research Experience and Significance	Not practicing	33	69.94	–	H= 0.34 P= 0.84
	Non-hospital-based practice setting	42	74.75	–	
	Hospital based practice setting	70	73.39	–	
Confidence in Conducting Research	Not practicing	31	65.85	–	H= 6.78 **P= 0.03***
	Non-hospital-based practice setting	42	83.68	–	
	Hospital based practice setting	68	65.51	–	
Research Outcomes	Not practicing	31	73.76	–	H= 1.02 P= 0.60
	Non-hospital-based practice setting	42	73.55	–	
	Hospital based practice setting	67	67.08	–	
Motivations for Future Research Plans	Not practicing	31	74.95	–	H= 2.50 P= 0.29
	Non-hospital-based practice setting	42	74.36	–	
	Hospital based practice setting	66	64.90	–	
**Years of work experience**					
Research Experience and Significance	None	29	75.38	–	H= 6.18 P= 0.19
	Less than 1 year	24	65.58	–	
	1-5 years	64	76.81	–	

	6-10 years	27	74.67	–	
	More than 10 years	2	19.50	–	
Confidence in Conducting Research	None	27	70.07	–	H= 0.43 P= 0.98
	Less than 1 year	24	73.38	–	
	1-5 years	62	70.18	–	
	6-10 years	27	73.44	–	
	More than 10 years	2	83.00	–	
Research Outcomes	None	27	73.06	–	H= 8.15 P= 0.09
	Less than 1 year	24	76.98	–	
	1-5 years	62	75.09	–	
	6-10 years	27	56.80	–	
	More than 10 years	1	2.00	–	
Motivations for Future Research Plans	None	27	79.09	–	H= 5.49 P= 2.4
	Less than 1 year	24	67.17	–	
	1-5 years	61	71.55	–	
	6-10 years	27	64.87	–	
	More than 10 years	1	6.50	–	
**URPC research focus**					
Research Experience and Significance	Clinical pharmacy and practice only	91	90.38	–	H= 3.02 P= 0.22
	Pharmaceutical sciences	25	83.38	–	
	Both	69	99.94	–	
Confidence in Conducting Research	Clinical pharmacy and practice only	89	85.96	–	H= 3.35 P= 0.19
	Pharmaceutical sciences	25	86.08	–	
	Both	67	99.53	–	
Research Outcomes	Clinical pharmacy and practice only	87	83.36	–	H= 3.34 P= 0.19
	Pharmaceutical sciences	25	91.94	–	
	Both	67	97.90	–	
Motivations for Future Research Plans	Clinical pharmacy and practice only	86	85.02	–	H= 3.32 P= 0.19
	Pharmaceutical sciences	25	83.10	–	
	Both	67	97.63	–	
**Additional research project taken**					
	Yes	114	97.28	11089.50	U= 3103.50

Research Experience and Significance	No	67	80.32	5381.50	**P= 0.01***
Confidence in Conducting Research	Yes	113	93.44	10558.50	U= 3114.50
	No	64	81.16	5194.50	P= 0.09
Research Outcomes	Yes	112	96.04	10756.00	U= 2740.00
	No	64	75.31	4820.00	**P= 0.01***
Motivations for Future Research Plans	Yes	111	95.43	10592.50	U= 2727.50
	No	64	75.12	4807.50	**P= 0.01***
**Publication**					
Research Experience and Significance	Yes	99	92.49	9156.50	U= 3614.50
	No	79	85.75	6774.50	P= 0.31
Confidence in Conducting Research	Yes	99	96.59	9562.00	U= 3209.00
	No	79	80.62	6369.00	**P= 0.03***
Research Outcomes	Yes	98	91.54	8970.50	U= 3622.50
	No	79	85.85	6782.50	P= 0.44
Motivations for Future Research Plans	Yes	97	85.66	8309.00	U= 3556.00
	No	79	91.99	7267.00	P= 0.36
**Participation in poster presentation**					
Research Experience and Significance	Yes	78	94.67	7384.50	U= 3340.50
	No	98	83.59	8191.50	P= 0.09
Confidence in Conducting Research	Yes	78	99.27	7743.00	U= 2982.00
	No	98	79.93	7833.00	**P= 0.01***
Research Outcomes	Yes	77	93.85	7226.50	U= 3322.50
	No	98	83.40	8173.50	P= 0.16
Motivations for Future Research Plans	Yes	76	89.55	6805.50	U= 3568.50
	No	98	85.91	8419.50	P= 0.60
**Participation in oral presentation**					
Research Experience and Significance	Yes	90	84.85	7636.50	U= 3541.50
	No	81	87.28	7069.50	P= 0.73
Confidence in Conducting Research	Yes	90	86.99	7829.50	U= 3555.50
	No	81	84.90	6876.50	P= 0.74
Research Outcomes	Yes	89	85.76	7633.00	U= 3581.00
	No	81	85.21	6902.00	P= 0.94
Motivations for Future Research Plans	Yes	88	86.01	7569.00	U= 3475.00
	No	81	83.90	6796.00	P= 0.76

* Indicates significance
